# Andrographolide Sulfonate Attenuates Acute Lung Injury by Reducing Expression of Myeloperoxidase and Neutrophil-Derived Proteases in Mice

**DOI:** 10.3389/fphys.2018.00939

**Published:** 2018-08-17

**Authors:** Fei Gao, Xing Liu, Ziying Shen, Xiaohui Jia, Han He, Jing Gao, Jianhong Wu, Chunhong Jiang, Hu Zhou, Yiping Wang

**Affiliations:** ^1^State Key Laboratory of Drug Research, Shanghai Institute of Materia Medica, University of Chinese Academy of Sciences, Shanghai, China; ^2^Department of Analytical Chemistry and CAS Key Laboratory of Receptor Research, Shanghai Institute of Materia Medica, University of Chinese Academy of Sciences, Shanghai, China; ^3^State Key Laboratory of Innovative Natural Medicine and TCM Injections, Ganzhou, China

**Keywords:** andrographolide sulfonate, acute lung injury, iTRAQ, quantitative proteomics, immunohistochemistry

## Abstract

Andrographolide sulfonate (Andro-S), a sulfonation derivative of andrographolide, is known to be effective in treating inflammation-related diseases, while the underlying mechanisms and global protein alterations in response to Andro-S remain unknown. This study aimed to investigate the pharmacological effects and potential targets of Andro-S in a murine model of acute lung injury (ALI). ALI was induced by aerosolized lipopolysaccharide (LPS) exposure before treatment with Andro-S. Inflammatory state of each treatment group was determined by histological analysis and quantification of inflammatory markers. Differentially expressed proteins in lung tissues were identified by an iTRAQ-based quantitative proteomic approach and further confirmed by immunohistochemistry analysis. Administration of Andro-S alleviated LPS-induced histological changes in the lung and reduced the expression of inflammatory markers in serum, bronchoalveolar fluid and lung tissues. Proteomic analysis identified 31 differentially expressed proteins from a total of 2,234 quantified proteins in the lung. According to bioinformatics analysis, neutrophil elastase (ELANE), cathepsin G (CTSG) and myeloperoxidase (MPO), three neutrophil-derived proteases related to immune system process and defense responses to fungi were chosen as potential targets of Andro-S. Further immunohistochemistry analysis confirmed the inhibitory effects of Andro-S on LPS-induced ELANE, CTSG and MPO up-regulation. These results indicate that Andro-S suppressed the severity of LPS-induced ALI, possibly by attenuating the expression of and neutrophil-derived proteases.

## Introduction

Acute lung injury (ALI), as a common complication of severe sepsis, trauma, and pneumonia, is associated with an increased risk of death and persistent hospitalization. *Andrographis paniculata Nees*, a traditional Chinese medicine and Indian medicine, is well known for its anti-inflammatory (Abu-Ghefreh et al., 2009), anti-viral (Gupta et al., 2017), and anti-bacterial activities (Banerjee et al., 2017). Preclinical evidence suggests that *A*. paniculata exerts potent anti-inflammatory effects, and benefits respiratory system through alleviation of upper respiratory tract infection.

The main bioactive constitute of *A*. paniculata is andrographolide, which has been shown to exert its anti-inflammatory effects through inhibition of NF-κB (Xia et al., 2004; Hidalgo et al., 2005; Zhu et al., 2013). However, the clinical use of andrographolide has been very limited owing to its poor solubility in water (Panossian et al., 2000; Ye et al., 2011; Bera et al., 2014). On the other hand, andrographolide sulfonate (Andro-S), the sulfonation product of andrographolide, is highly water-soluble and has been widely used in treating inflammation-related disease, including bronchitis, tonsillitis, and bacillary dysentery. More recent studies have revealed that Andro-S alleviates acute inflammation more potently than andrographolide (Peng et al., 2016). Although some studies have investigated the mechanisms of Andro-S during recent decades, few have provided information about global protein alterations in cells or tissues in response to Andro-S. Thus, elucidating key biological processes in which Andro-S is involved and identifying potential targets of Andro-S *in vivo* remain a significant challenge.

Proteomic technologies for large-scale qualitative and quantitative analysis have matured over the past two decades (Ong and Mann, 2005; Gstaiger and Aebersold, 2009; Zhang et al., 2013; Zhou et al., 2013). Most proteomic studies employ a bottom-up protocol in which proteins are first digested into peptides. After chromatographic separation, these peptides are detected using electrospray ionization-tandem mass spectrometry (MS/MS), and then the corresponding proteins are identified through database-searching algorithms (Gygi et al., 1999; Washburn et al., 2001; Mann and Kelleher, 2008). A number of stable isotope labeling-based quantitative proteomic techniques have been developed (Gygi et al., 1999; Yao et al., 2001; Ong et al., 2002; Ross et al., 2004; McClatchy et al., 2007; Ow et al., 2008). One such method is iTRAQ (isobaric tags for relative and absolute quantitation) which identifies proteins from peptide fragments and quantifies proteins from low-mass reporter ions at the MS/MS level. Because of its ability to identify low-abundance proteins or those with molecular masses that are too small or too large, iTRAQ has been extensively applied in the life sciences for quantitative proteomics.

Herein, using a murine model of ALI, we show that Andro-S potently inhibited pulmonary inflammation induced by lipopolysaccharide (LPS) inhalation. We further utilized an iTRAQ-based quantitative proteomic approach to gain insight into possible mechanisms-of-action of Andro-S by identifying differentially expressed proteins in lung tissues. In our analysis, 2,234 proteins were quantified across all of the 25 samples, and 31 proteins were significantly changed under a strict data filtration criterion, including three immune response-related proteins (ELANE, CTSG, and MPO). Further biological validation experiments confirmed that Andro-S can inhibit the LPS-induced up-regulation of ELANE, CTSG, and MPO.

## Methods

### Reagents

Andro-S was provided by Jiangxi Qingfeng Pharmaceutical Co. Ltd., Ganzhou, China. LPS (*Escherichia coli* 055: B5, L2880) was purchased from Sigma-Aldrich (St Louis, MO, USA). Enzyme-linked immunoassay (ELISA) kits for mouse IL6 (interleukin-6) and TNFα (tumor necrosis factor-α) were purchased from R&D Systems (Minneapolis, MN, USA).

### Murine model ALI

Male C57BL/6J mice (19–26 g) were purchased from Shanghai Center of Experimental Animals, Chinese Academy of Sciences. The Animal Care and Use Committee of Shanghai Institute of Materia Medica, Chinese Academy of Sciences approved the experimental procedure used in this study. All mice were housed in a temperature-controlled room under a 12-h light-dark cycle with free access to standard chow and water.

Mice were randomly divided into five groups: control group (*n* = 5), blank group (saline + Andro-S 50 mg/kg, *n* = 5), model group (LPS + saline, *n* = 5), low-dose treatment group (LPS + Andro-S 10 mg/kg, *n* = 5), and high-dose treatment group (LPS + Andro-S 50 mg/kg, *n* = 5).

ALI was induced by exposing mice to aerosolized LPS (2.5 mg/mL) for 30 min twice at a 2-h interval; mice in the blank group were exposed to saline. LPS was dissolved in saline and aerosolized with an air nebulizer. Immediately after the second exposure, saline or Andro-S (10 mg/kg or 50 mg/kg) was administered by intravenous injection. Five hours after treatment, mice were anesthetized with an intraperitoneal injection of sodium pentobarbital (40 mg/kg), and blood samples were collected from the ventral aorta and centrifuged to obtain serum.

For both pathological studies and proteomic analyses, two sets of animal experiments were required to collect all tissue samples. In one set of animals (*n* = 5 per group), the left lungs were ligated and lavaged to obtain bronchoalveolar lavage fluid, and the right lungs were harvested and fixed for histological evaluation. In another set of animals (*n* = 5 per group), lungs were perfused with heparinized saline, harvested and stored at −80°C for proteomic studies.

### Bronchoalveolar lavage fluid preparation

For bronchoalveolar lavage fluid (BALF) preparation, the right lung was ligated, and the upper part of the trachea was cannulated. Then, 0.5 mL of phosphate-buffered saline (PBS) was infused intratracheally into the left lung and gently aspirated. This procedure was repeated three times, resulting in a total BALF volume of 1.5 mL. After centrifuging BALF for 5 min at 300 × g, supernatants were collected and stored at−80°C. The right lower lobe of the lung was harvested and fixed in 10% formalin for histology.

### ELISA for IL6 and TNFα

The concentrations of IL6 and TNFα, indicators of an inflammatory state, were measured in BALF and serum samples using an ELISA kit according to the manufacturer's protocol (R&D Systems).

### Histopathology and immunohistochemistry (IHC) analysis

For histopathological analyses, lung tissues (*n* = 5 per group) were fixed, dehydrated, paraffin-embedded, cut into 5-μm sections, and stained with hematoxylin and eosin (H&E). A lung injury score was calculated by evaluating five parameters (neutrophils in the alveolar space, neutrophils in the interstitial space, hyaline membranes, proteinaceous debris filling the airspaces and alveolar septal thickening) based on a scoring system developed by the American Thoracic Society (Matute-Bello et al., 2011). A total of 20 fields of views were examined for each lung section.

For IHC analyses, paraffin-embedded sections were deparaffinized in xylene and rehydrated. After antigen retrieval with sodium citrate buffer, slides were treated with 3% H_2_O_2_ to quench endogenous peroxidase activity. Non-specific antibody binding was blocked by incubating slides with animal-free blocking solution (Cell Signaling Technology, #15019), after which slides were immunostained for the following proteins using the indicated primary antibodies: IL6 (1:200; Abcam, #ab6672), TNFα (1:100; Abcam, #ab6671), ELANE (1:100; Abcam, #ab68672), CTSG (1:400; Bioryt, #or10253) and MPO (1:100; Invitrogen, #PA5-16672). Antibody-antigen complexes were detected and visualized using horseradish peroxidase (HRP)-conjugated IHC Detection Reagent (Cell Signaling Technology, #8114) and a DAB substrate kit (Cell Signaling Technology, #8059). The slides were then counterstained with Mayer's hematoxylin and mounted with DPX. Stained slides were photographed and analyzed using Image-Pro software. The mean intensity of five non-overlap fields was averaged for each stained slide.

### iTRAQ-based proteomic analysis

#### Protein extraction and digestion

Lung tissues were washed several times with ice-cold PBS to completely remove residual blood, then homogenized and lysed in SDT buffer (100 mM dithiothreitol [DTT], 4% sodium SDS, 100 mM Tris-HCl, pH 7.6). Protein concentration was determined using a tryptophan-fluorescence assay (Wisniewski, 2013), and confirmed by sodium dodecyl sulfate-polyacrylamide gel electrophoresis (SDS-PAGE) on a 12% gel. An equal amount of protein (50 μg) from each sample was digested using the filter-aided sample preparation (FASP) procedure (Wisniewski et al., 2009), and the digested samples were evaporated to dryness in a Speed-Vac sample concentrator.

#### iTRAQ labeling and high-pH reversed-phase HPLC fractionation

Dried peptides were reconstituted in 100 mM triethylammonium bicarbonate (TEAB) and processed with 8-plex iTRAQ reagent (Applied Biosystems, Foster City, CA, USA) according to the manufacturer's protocol. Briefly, one unit of iTRAQ reagent was thawed at room temperature and reconstituted in 50 μL isopropanol. Peptides were labeled with the isobaric tags and incubated at room temperature for 2 h. Samples were combined and the labeling reaction was stopped by adding 100 μL of ddH_2_O. The samples were evaporated to dryness in a vacuum concentrator.

The labeled peptide mixture was fractionated by high-performance liquid chromatography (HPLC) on a Waters XBridge BEH130 C18 column (4.6 × 250 mm, 3.5 μm particle size) using an Ultimate 3000 HPLC system (Agilent, USA) operating at 1 mL/min. Mobile phase A consisted of 10 mM ammonium formate containing 90% acetonitrile; both buffers were adjusted to pH 10 with ammonium hydroxide, as described previously (Batth et al., 2014). The fractionation gradient was as follows: 1% B to 25% B in 50 min, 60% B in 4 min, and ramped to 70% B over 2 min. At this point, fraction-collection was halted, and the gradient was held at 70% B for 5 min before being ramped back to 1% B, at which point the column was washed and equilibrated. All fractions were collected manually; 28 fractions were collected for each sample and concatenated to 14 (pooling equal interval RPLC fractions). The fractioned samples were evaporated to dryness in a Speed-Vac sample concentrator and stored at −80°C until LC-MS/MS analysis.

#### LC-MS/MS analysis

The LC-MS/MS analyses were performed as previous described, with some modifications (Liu et al., 2016; Chen et al., 2017). The resulting peptides were re-suspended in 10 μL of water containing 0.1% formic acid. A 4-μL volume of the peptide solution was injected onto a 75 μm × 150 mm fused silica pre-column packed in-house with 3 μm ReproSil-Pur C18 beads (120 Å; Dr. Maisch GmbH, Ammerbuch, Germany) using an Easy nano-UPLC1000 system (Thermo Electron, Waltham, MA, USA). Mobile phase A consisted of 0.1% formic acid and mobile phase B consisted of 0.1% formic acid in acetonitrile. The peptides were eluted using a gradient (5–80% acetonitrile containing 0.1% formic acid) at a flow rate of 300 nL/min over a 200-min period. Data were acquired using a nano-ESI quadrupole Exactive (Q Exactive) mass spectrometer (Thermo Electron). The mass spectrometer was operated in data-dependent mode with each full MS scan, followed by MS/MS for the 15 most intense ions, using the following parameters: precursor ion charge, ≥+2; precursor ion isolation window, 2 Da; normalized HCD collision energy, 33%; dynamic exclusion, 30 s. A full MS scan was collected for peptides from 100 to 1,700 m/z on the Orbitrap analyzer at a resolution of 70,000 (at m/z = 200), and subsequent MS/MS analyses were performed in the Orbitrap analyzer at a resolution of 17,500 (at m/z = 200).

#### LC-MS/MS data analysis

Maxquant (Cox and Mann, 2008) (http://maxquant.org/, version 1.3.0.5) was used to generate peak lists from raw files, and Andromeda (Cox et al., 2011) was used to search the protein sequence database. Report ion type was chosen, and 8-plex iTRAQ was set as the isobaric labels. Carbamidomethyl (C) was set as a fixed modification, and oxidation (M, +15.99492 Da) was set as a variable modification. Acquired MS/MS spectra were searched against a decoyed proteome sequence of mouse (Mus musculus C57BL/6J; UP000000589; UniProt proteome database). The precursor mass tolerances for the first and main searches were set at 20 and 6 ppm, respectively, and the fragment mass tolerance for HCD MS/MS spectra was set at 20 ppm. Trypsin/P was selected as the digestive enzyme with allowance for two potential missed cleavages. The false-discovery rate (FDR) for peptides and protein groups was rigorously controlled to below 1%. FDR was calculated as the number of hits from the reverse database divided by the number of forward hits (Cox et al., 2011). Each confident protein assignment was based on at least one unique peptide. The R software package was used for normalizing report ion intensities and for calculating *p*-value between each group using two-tailed Student's *t*-test. Identified differentially expressed proteins were functionally annotated using DAVID software 6.8 (http://david.abcc.ncifcrf.gov/) (Dennis et al., 2003). The protein-protein interaction network was created using String Consortium software (version 10.5; http://string-db.org) (Franceschini et al., 2013).

### Statistical analysis

Data are expressed as means ± standard deviation (SD). For the proteomic datasets, the significantly changed proteins between two groups were analyzed using two-tailed Student's *t*-test, and one-way analysis of variance (ANOVA) followed by Tukey test correction for multiple treatment groups was also calculated for selecting the differentially expressed proteins among the different groups. For the immunohistochemistry experiments, one-way analysis of variance (ANOVA) followed by Newman-Keuls test correction were used to demonstrate the significant difference from multiple treatment groups. A *P*-value < 0.05 was considered statistically significant.

## Results

### Andro-S attenuates ALI *in vivo*

**Figure 1:**
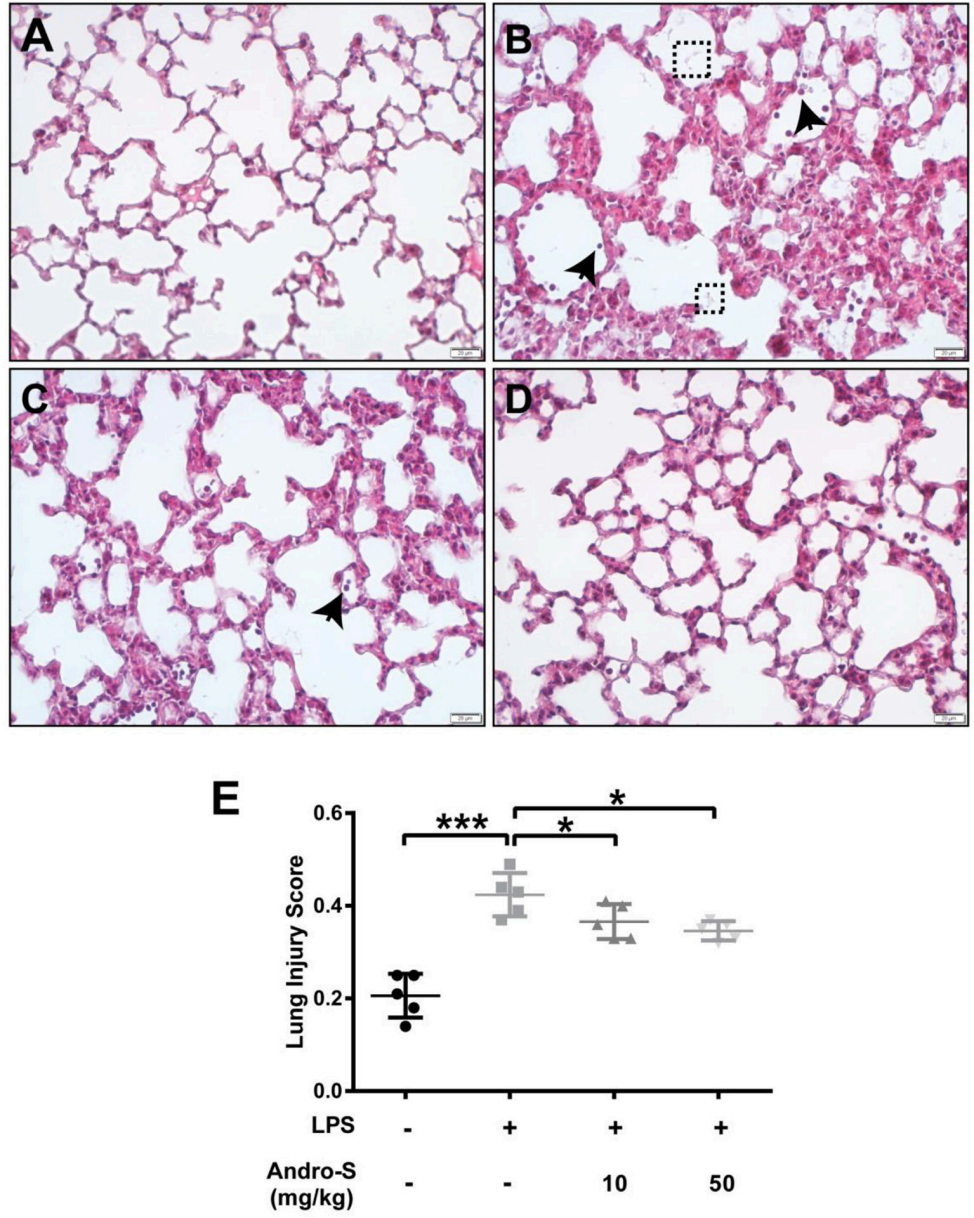
Andro-S treatment alleviates LPS-induced histological lung injury. Mice were challenged with LPS twice for 30 min at a 2-h interval and sacrificed 5 h later. A low dose or high dose of Andro-S was administered immediately after the second LPS exposure. **(A–D)** Representative histological images of lungs from mice treated as indicated (**A**, control; **B**, model; **C**, low-dose treatment; **D**, high-dose treatment). Arrows indicate neutrophils. Dashed boxes indicate proteinaceous debris. Original magnification, × 400. Scale bar represents 20 μm. **(E)** Quantification of mean lung injury score for each treatment group. Values are mean ± SD (*n* = 5 per group; ^*^P < 0.05, ^***^P < 0.001 vs. model group by one-way ANOVA with Newman-Keuls multiple comparisons test).

A histological analysis of lungs after LPS exposure revealed neutrophils infiltration, alveolar wall thickening and the presence of proteinaceous debris (Figure 1B), all of which were absent in the control group (Figure 1A). Intravenous injection of 10 mg/kg or 50 mg/kg Andro-S decreased the number of neutrophils and partially reversed the alveolar thickening induced by LPS (Figures 1C,D), and thus significantly decreased lung injury score (Figure 1E).

### Andro-S treatment inhibits cytokine production *in vivo*

To further characterize the anti-inflammatory effects of Andro-S, we evaluated the levels of two pro-inflammatory cytokines, IL6 and TNFα in both serum and BALF samples. As shown in Table 1, LPS challenge caused a significant increase in IL6 and TNFα protein levels in both serum and BALF compared with controls, indicating the presence of both systemic inflammation and local pulmonary inflammation. IL6 and TNFα concentrations increased much more robustly in BALF than in serum reflecting direct stimulation of the lung by inhaled LPS. Andro-S treatment after LPS exposure significantly attenuated increases in IL6 and TNFα protein levels in serum. The inhibitory effects of Andro-S on the production of cytokines were less striking in BALF than in serum. Low-dose and high-dose Andro-S treatment decreased BALF TNFα level by 31.4 and 42.4%, respectively, relative to that in the model group. BALF IL6 level were not significantly different between the model group and Andro-S treatment groups (Table 1).

**Table 1:** Inflammatory Markers in serum and BALF samples.

	**Serum (pg/mL)**		**BALF (pg/mL)**	
**Treatments**	**TNFα**	**IL6**	**TNFα**	**IL6**
Control	0 ± 0	5.04 ± 6.44	62.42 ± 2.42	0 ± 0
Model	13.43 ± 7.6	151.66 ± 54.46	696.22 ± 128.51	1029.23 ± 157.55
Low dose	3.78 ± 0.84^**^	65.33 ± 33.99^**^	477.71 ± 64.81^**^	924.47 ± 195.25
High dose	0.99 ± 0.64^***^	35.69 ± 14.49^***^	401.3 ± 133.55^***^	890.97 ± 137.57

*Values are mean ± SD (n = 5 per group)*.

** *P < 0.01*,

*** *P < 0.001 vs. model group by one-way ANOVA with Newman-Keuls multiple comparisons test*.

**Figure 2:**
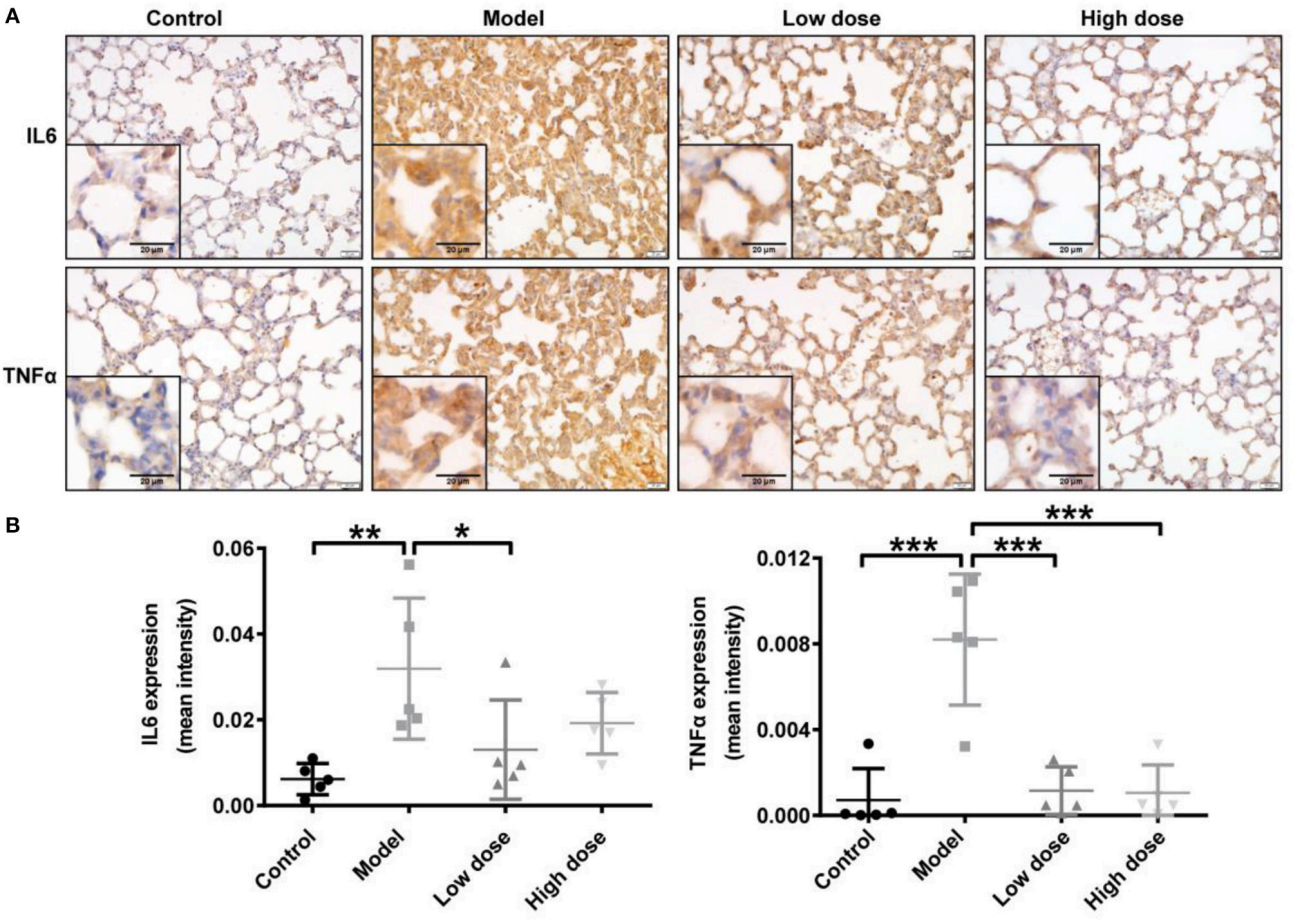
Effects of Andro-S on IL6 and TNFα expression in lung tissue. **(A)** IL6 and TNFα staining in lung sections of each group. Original magnification, × 400. Scale bar represents 20 μm. **(B)** Quantification of IL6 and TNFα immunohistochemistry in lung tissue sections from each group. Values are mean ± SD (*n* = 5 per group; ^*^P < 0.05, ^**^P < 0.01, ^***^P < 0.001 vs. model group by one-way ANOVA with Newman-Keuls multiple comparisons test).

Consistent with the cytokine levels in serum and BALF, representative IHC images showed markedly enhanced IL6 and TNFα immunostaining in the cytoplasm of cells in the lung interstitial region after LPS inhalation compared with that in healthy control mice. TNFα expression was significantly decreased after Andro-S treatment compared with the model group; IL6 expression also trended slightly lower, although this decrease did not reach statistical significance (Figure 2).

### iTRAQ-based proteomics identification of differentially expressed proteins in lung tissue sections from different treatment group

**Figure 3:**
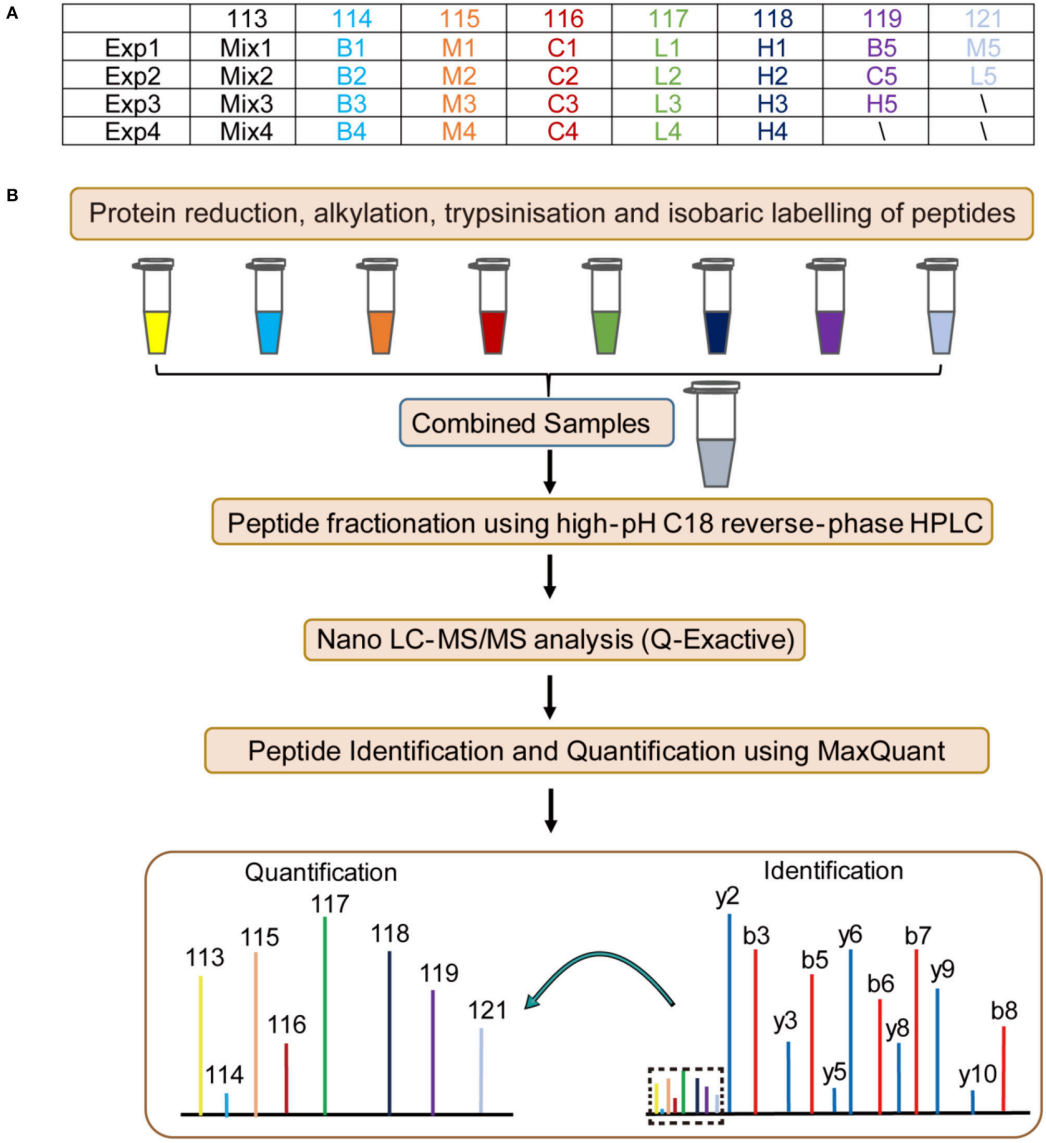
Flow chart of the proteomic analysis. B: blank group (saline +Andro-S 50 mg/kg); C: control group (saline + saline); M: model group (LPS + saline); L: low-dose treatment group (LPS + Andro-S 10 mg/kg); H: high-dose treatment group (LPS + Andro-S 50 mg/kg). **(A)** Experimental design for the iTRAQ-labeling strategy. **(B)** Workflow of the iTRAQ-based proteomics analysis.

To elucidate the probable mechanism of Andro-S effects on pulmonary inflammation, we designed four independent 8-plex iTRAQ experiments on lung tissue sections from five different groups: blank group, control group, model group, low-dose treatment group, and high-dose treatment group. Details of the labeling experimental design are shown in Figure 3A. Sample pooling in combination with biological replicates made it possible to average the biological variability of several samples within a single isobaric tag experiment. As shown in Figure 3B, the resulting protein extracts were digested with trypsin and subsequently labeled using iTRAQ chemistry, providing relative quantitation of protein expression. Pooled peptides from each experiment were then separated into 14 fractions by high-pH C18 reverse-phase liquid chromatography, followed by Nano LC-ESI-MS/MS analysis with a Q-Exactive mass spectrometer. Fragment spectra were obtained with high mass accuracy using higher energy collisional dissociation (HCD). The reporter ion intensity of each protein across individual samples was obtained using MaxQuant (Cox and Mann, 2008).

A total of 2,630,773 MS spectra were obtained from the iTRAQ-LC-MS/MS proteomic analyses of all 25 lung tissue samples. To provide highly confident protein assignments, we used a stringent FDR of <1% for filtering peptide and protein data. After strict data filtering, 324,595 unique spectra corresponding to 4,666 unique proteins were identified.

**Figure 4:**
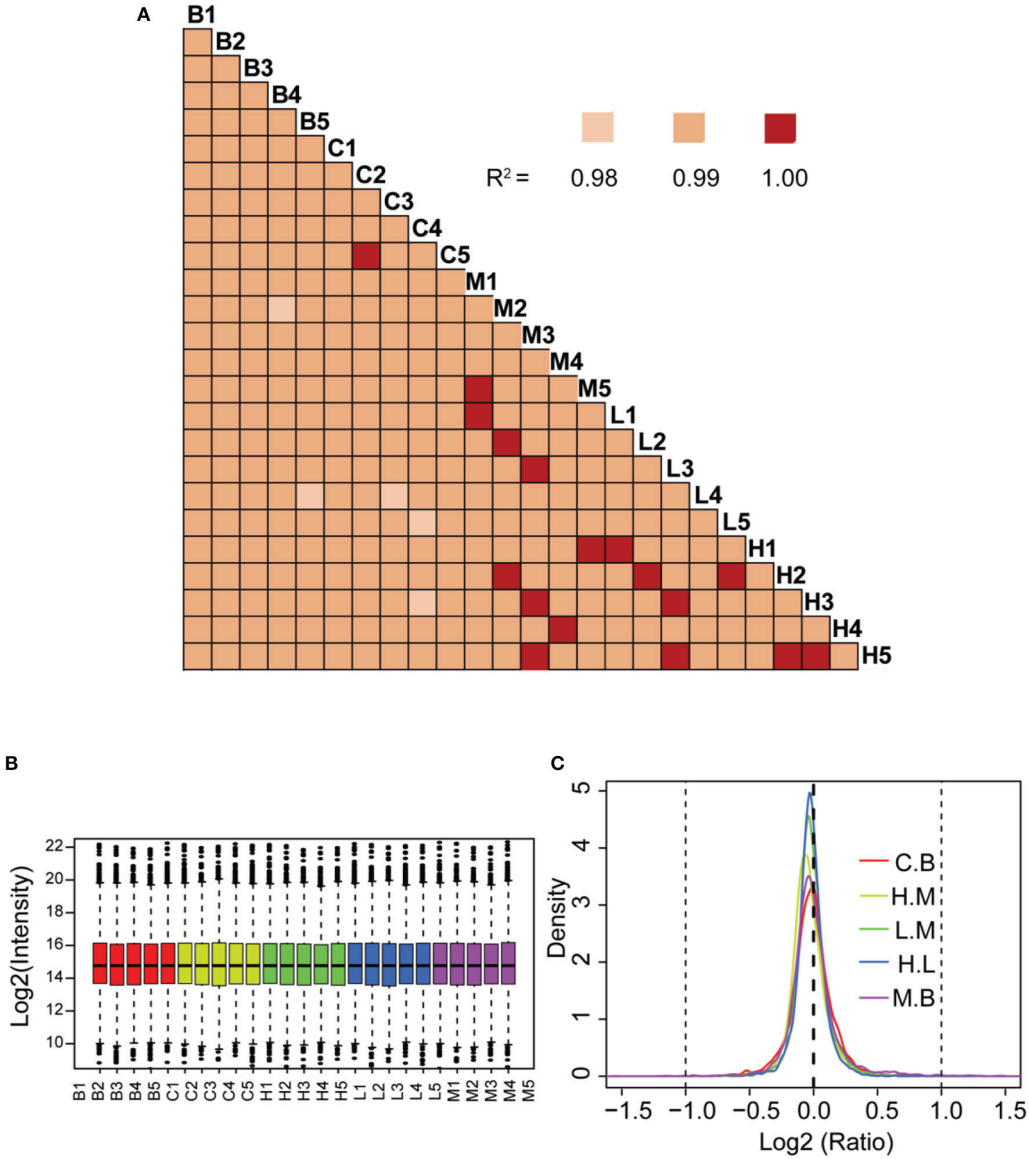
Quantitative proteomic analysis of lung tissue sections from different treatment groups. **(A)** Correlation analysis between any two biological replicates. **(B)** Box plots analysis of all 25 samples. **(C)** Density plot of log-transformed ratios of any two groups. B: blank group (saline +Andro-S 50 mg/kg); C: control group (saline + saline); M: model group (LPS + saline); L: low-dose treatment group (LPS + Andro-S 10 mg/kg); H: high-dose treatment group (LPS + Andro-S 50 mg/kg).

The correlation co-efficient of the iTRAQ reporter ion intensities between any two cohorts was greater than 0.99 (Figure 4A), demonstrating good quantitative reproducibility of the iTRAQ experiment. A boxplot analysis further showed similar average reporter ion intensities across samples (Figure 4B). The density plots of log_2_ ratios of any two groups were very similar to a normal distribution (Figure 4C). Box plots and density plots indicated that iTRAQ experimental procedures showed no bias toward different samples. We also evaluated the coefficient of variation (CV) of all identified proteins in each group. This analysis showed that the SD for most proteins was less than 0.2, revealing that most identified proteins were unchanged and the quantitative accuracy of the experiment was high (Figure S1).

In our dataset, 2,958 proteins detected in at least three replicates were designated as quantified proteins. Of those, 2,234 were quantified across all of the 25 samples, indicating that each protein generated expression information for different treatment statuses (Table S1). We also performed *t*-test analysis between any two groups to identify differentially expressed proteins that were potential targets of Andro-S. To determine potential targets of Andro-S, we filtered proteins according to the following four criteria (Smith et al., 2015; Xia et al., 2017; Zhang et al., 2017; Zhou et al., 2017): (1) significant over- or under-expression in the model group relative to the control group (Student's *t*-test *P* < 0.05); (2) significant over- or under-expression in high-dose treatment group compared with the model group (Student's *t*-test *P* < 0.05); (3) no significant changes in expression levels in the control group compared to the blank (Student's *t*-test *P* > 0.05) and (4) model group vs. control group or high-dose treatment group vs. model group (one-way analysis of variance (ANOVA) followed by Tukey test correction *P* < 0.05). A total of 31 proteins were identified as potential differentially expressed proteins based on these criteria (Table 2).

**Table 2:** List of the 31 differentially expressed proteins.

**Gene name**	**Protein description**	**Ratio M/C**	**Ratio H/M**	**Ratio L/M**	**Ratio C/B**	**Two-tailed Student's** ***t*****-test**			**One way ANOVA followed by Tukey's test**			
						***P*-value M.C**	***P*-value H.M**	***P*-value C.B**	**P.anov. test**	***P*-value M.C**	***P*-value H.M**	***P*-value C.B**
Mpo	Myeloperoxidase	1.90	0.82	0.85	0.99	7.79E-05	2.66E-02	8.00E-01	5.55E-09	5.57E-08	1.16E-02	1.00E+00
Pkm	Pyruvate kinase PKM	1.13	0.88	0.90	1.02	3.42E-02	2.88E-02	6.57E-01	2.38E-02	3.47E-02	3.31E-02	9.94E-01
Ngp	Neutrophilic granule protein	3.42	0.74	0.82	1.11	2.19E-05	4.92E-02	2.87E-01	4.35E-09	3.84E-08	1.31E-02	9.92E-01
Ltf	Lactotransferrin	1.98	0.78	0.83	1.02	6.12E-04	2.76E-02	5.99E-01	1.14E-08	6.04E-08	2.77E-03	1.00E+00
Ncl	Nucleolin	1.55	0.84	0.86	1.17	4.02E-04	4.35E-02	6.59E-02	6.91E-05	2.99E-05	5.37E-02	3.12E-01
S100a9	Protein S100-A9	2.76	0.80	0.86	1.04	8.89E-06	3.43E-02	6.96E-01	1.66E-10	3.12E-09	1.42E-02	9.99E-01
Itgam	Integrin alpha-M	1.65	0.85	0.88	0.93	6.57E-05	2.62E-02	2.65E-01	2.21E-08	5.60E-07	2.68E-02	8.77E-01
Ptprc	Receptor-type tyrosine-protein phosphatase C	1.30	0.87	0.91	0.95	1.98E-02	3.23E-02	6.14E-01	5.61E-04	3.49E-03	1.49E-01	9.44E-01
Cd177	CD177 antigen	1.79	0.84	0.86	0.94	3.47E-05	2.58E-02	4.19E-01	1.37E-08	2.56E-07	3.42E-02	9.58E-01
Sun2	SUN domain-containing protein 2	1.21	0.89	0.92	0.98	1.77E-03	3.62E-02	6.06E-01	2.89E-03	8.29E-03	1.75E-01	9.93E-01
Cybb	Cytochrome b-245 heavy chain	1.68	0.84	0.86	0.97	7.15E-05	3.00E-02	6.46E-01	1.69E-08	1.85E-07	1.57E-02	9.96E-01
S100a8	Protein S100-A8	3.09	0.77	0.80	1.00	1.04E-05	3.01E-02	9.45E-01	6.89E-11	9.53E-10	4.38E-03	1.00E+00
Pgd	6-phosphogluconate dehydrogenase	1.16	0.89	0.92	1.00	1.45E-02	3.15E-02	9.54E-01	9.95E-03	1.41E-02	5.57E-02	1.00E+00
Csrp1	Cysteine and glycine-rich protein 1	1.16	0.85	0.85	0.96	3.89E-02	1.82E-02	4.75E-01	5.86E-03	3.87E-02	1.82E-02	9.31E-01
Coro1a	Coronin-1A; Coronin	1.48	0.79	0.86	1.01	1.03E-03	1.45E-02	8.66E-01	1.79E-05	3.86E-05	5.43E-03	1.00E+00
Arhgdib	Rho GDP-dissociation inhibitor 2	1.47	0.81	0.88	0.94	1.64E-03	2.20E-02	3.97E-01	7.42E-06	6.62E-05	1.40E-02	9.28E-01
Mmp9	Matrix metalloproteinase-9	1.94	0.81	0.82	0.96	7.21E-07	9.04E-03	5.66E-01	2.89E-09	3.19E-08	7.90E-03	9.95E-01
Hmgn2	Non-histone chromosomal protein HMG-17	1.48	0.81	0.88	1.12	6.45E-04	1.41E-02	2.32E-01	4.25E-04	2.79E-04	4.38E-02	6.64E-01
Srsf7	Serine/arginine-rich splicing factor 7	1.58	0.85	0.87	1.10	3.97E-04	2.96E-02	3.84E-01	4.74E-05	5.03E-05	1.10E-01	8.09E-01
Ctsg	Cathepsin G	2.22	0.78	0.84	0.94	1.96E-04	3.85E-02	6.44E-01	5.30E-08	7.20E-07	2.57E-02	9.93E-01
Elane	Elane	1.62	0.82	0.91	1.00	3.37E-03	3.74E-02	9.27E-01	3.74E-04	1.50E-03	2.24E-01	1.00E+00
Cd44	CD44 antigen	1.38	0.87	0.89	0.98	1.11E-03	1.29E-02	7.74E-01	1.03E-05	4.85E-05	4.89E-02	9.97E-01
Cotl1	Coactosin-like protein	1.18	0.87	0.93	1.03	1.31E-02	4.33E-03	5.58E-01	3.70E-03	5.04E-03	1.74E-02	9.71E-01
Lcn2	Neutrophil gelatinase-associated lipocalin	2.45	0.86	0.86	1.01	8.59E-06	3.83E-02	9.38E-01	1.02E-11	3.28E-10	4.82E-02	1.00E+00
Tra2b	Transformer-2 protein homolog beta	1.41	0.84	0.88	1.10	1.07E-03	1.83E-02	1.38E-01	1.29E-05	6.88E-06	5.95E-03	4.45E-01
G6pdx	Glucose-6-phosphate 1-dehydrogenase X	1.28	0.84	0.92	0.95	2.28E-03	1.65E-02	5.18E-01	1.25E-03	7.50E-03	6.97E-02	9.54E-01
Chi3l1	Chitinase-3-like protein 1	1.32	0.82	0.88	0.91	3.30E-02	3.81E-02	4.40E-01	1.78E-03	1.18E-02	7.53E-02	8.51E-01
RBM8	RNA-binding protein 8A	1.18	0.91	0.94	1.02	8.35E-03	2.11E-02	7.29E-01	2.53E-02	2.79E-02	3.84E-01	9.98E-01
Itgb2l	Integrin beta-2-like protein;Integrin beta	1.66	0.88	0.86	0.87	1.75E-03	4.29E-02	3.21E-01	3.46E-07	1.56E-05	3.03E-01	7.06E-01
Marcksl1	MARCKS-related protein	1.48	0.79	0.83	0.94	3.02E-03	2.95E-02	5.52E-01	2.34E-03	6.92E-03	1.14E-01	9.85E-01

*B, blank group (saline +Andro-S 50 mg/kg); C, control group (saline + saline); M, model group (LPS + saline); L, low-dose treatment group (LPS + Andro-S 10 mg/kg); H, high-dose treatment group (LPS + Andro-S 50 mg/kg)*.

### Functional analysis of differentially expressed proteins

**Figure 5:**
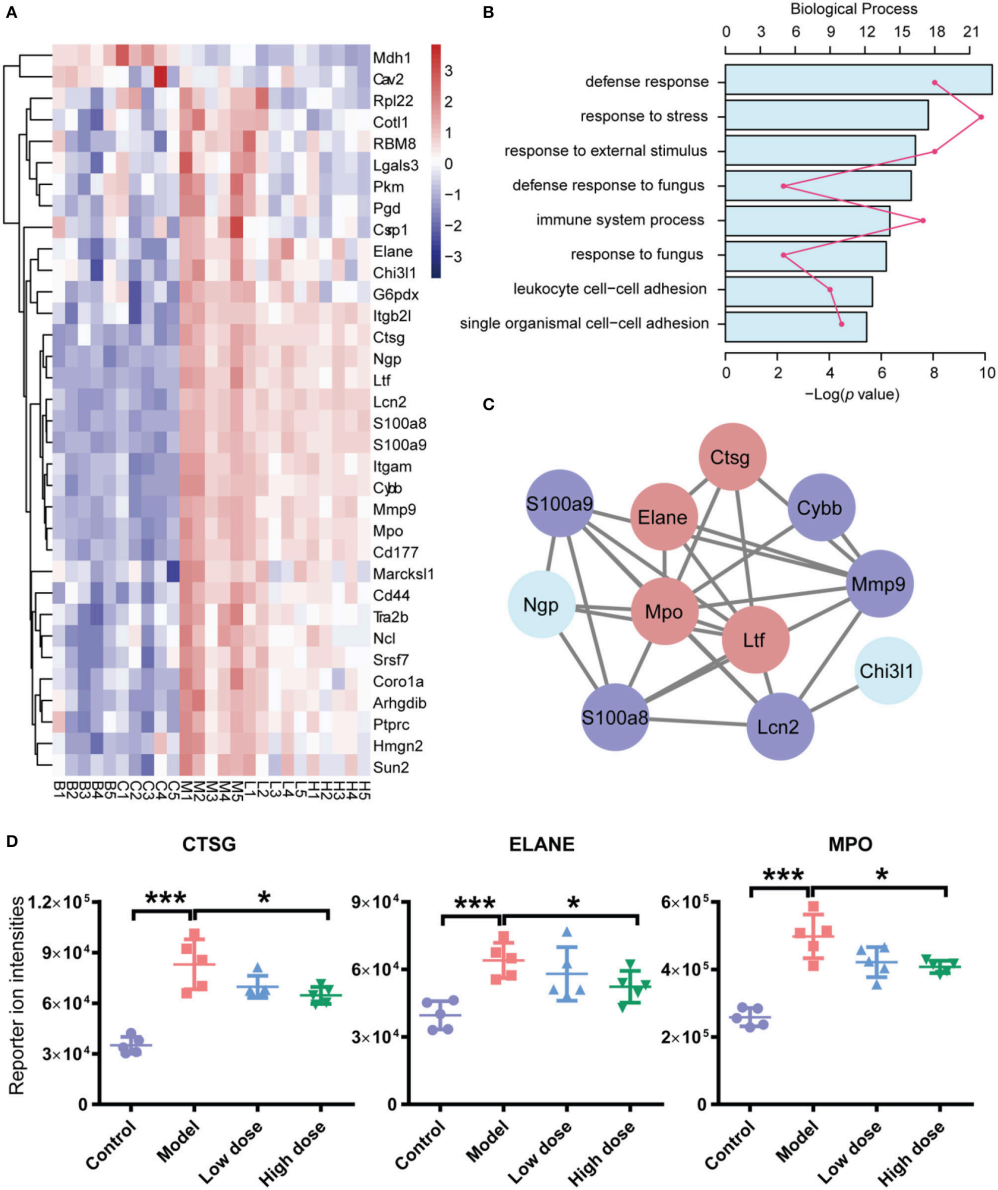
Functional analyses of differentially expressed proteins (DEPs). **(A)** Hierarchical clustering analysis of five groups. **(B)** Functional classification of DEPs based on biological process GO annotation terms. **(C)** PPI network analysis of PPIs. There are 11 significant proteins involved in the PPIs network. Red nodes represent proteins involved in immune system processes, and blue nodes represent proteins involved in defense responses against fungi. **(D)** iTRAQ reported ion intensities of CTSG, ELANE, and MPO. Values are mean ± SD (*n* = 5 per group). ^*^P < 0.05, ^***^P < 0.001 vs. model group by two-tailed Student's *t*-test test.

To discern the proteomic changes, we prepared a heat-map of the 31 protein intensities, as shown in Figure 5A. This figure shows that expression levels of many proteins were elevated in the model group compared with the control group. After treatment with Andro-S (both low dose and high dose), expression levels of these proteins decreased.

To better understand the probable roles of these differentially expressed proteins, we performed a Gene Ontology (GO) categorization analysis using DAVID Bioinformatics Resources. All 31 differentially expressed proteins were annotated with respect to cellular compartments and molecular functions. Each identified protein was associated with at least one annotation term within the GO cellular component, biological process and molecular categories, reflecting the fact that individual proteins are usually involved in multiple molecular and cellular functions. The major biological process categories were defense response, response to stress, and response to external stimulus (Figure 5B). The most common molecular functions were glycosaminoglycan binding, heparin binding, and sulfur compound binding (Figure S2A). The major cellular component categories were vesicle, membrane-bounded vesicle, and extracellular exosome (Figure S2B).

In addition, we searched for known and predicted interactions for the differentially expressed proteins identified by iTRAQ-based proteomics in the STRING protein-protein interaction database (http://string-db.org), and constructed a protein-protein interaction (PPI) network (Figure 5C). Among the 31 significantly changed proteins, 11 were included in the network. We further combined the biological process category results of GO annotation with the PPI network. Immune system process and defense response to fungus were the categories most closely related to the pharmacological effects of Andro-S. Red nodes in Figure 5C represent proteins involved in immune system processes, and blue nodes represent proteins involved in defense responses against fungi. Interestingly, four proteins, ELANE (neutrophil elastase), CTSG (cathepsin G), MPO (myeloperoxidase), and LTF (lactotransferrin), were involved in both immune system process and defense responses to fungi. Considering the essential role of enzymes in immune responses to inflammatory insults, we selected ELANE, CTSG, and MPO for further biological validation.

### Confirmation of differentially expressed proteins by IHC

**Figure 6:**
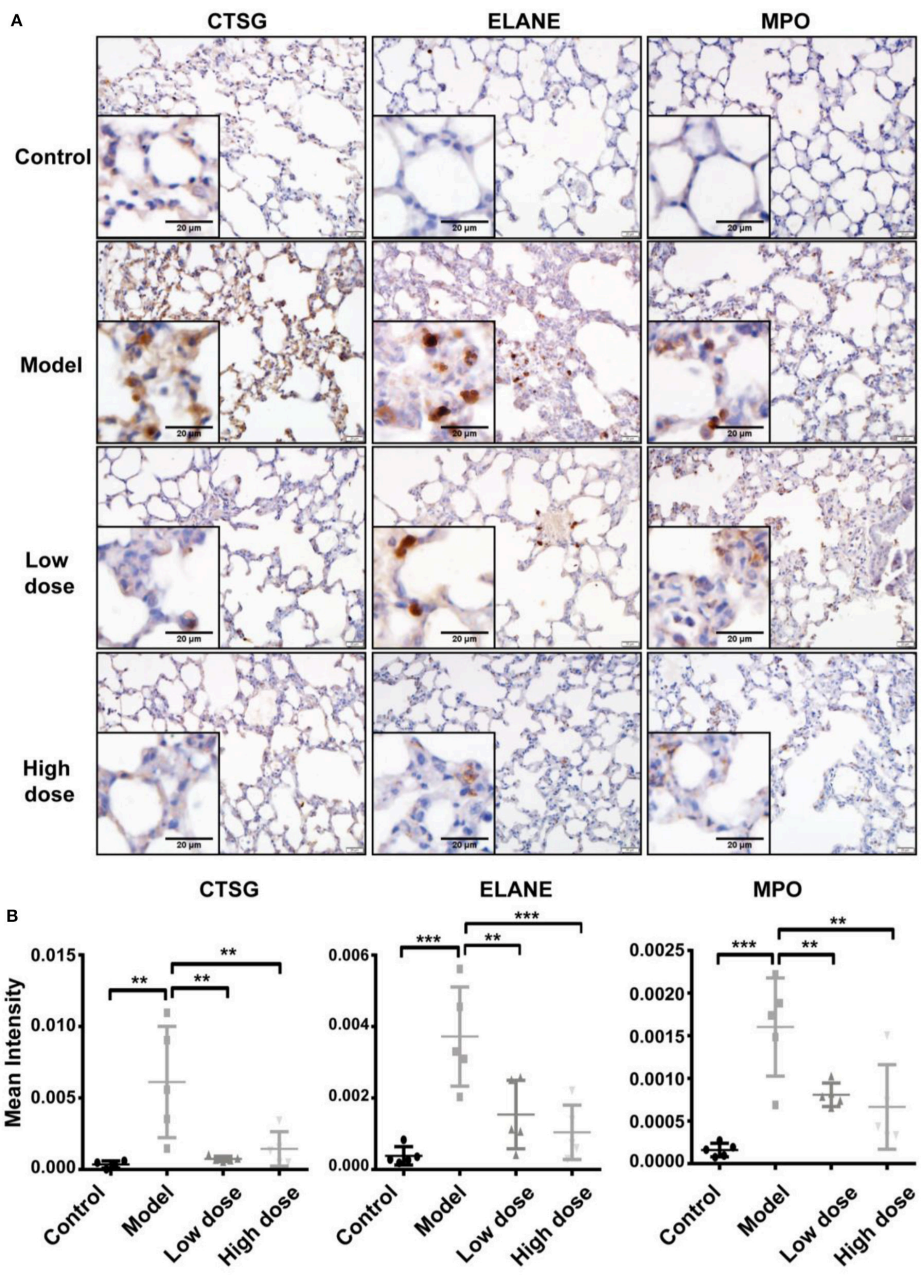
Validation of CTSG, ELANE, and MPO protein levels. **(A)** Representative images of CTSG, ELANE, and MPO immunostaining in the lung. Original magnification, × 400. Scale bar represents 20 μm. **(B)** Quantification of CTSG, ELANE, and MPO immunostaining. Values are mean ± SD (*n* = 5 for each treatment group. ^**^P < 0.01, ^***^P < 0.001 vs. model group; one-way ANOVA with Newman-Keuls multiple comparisons test).

The three enzymes, CTSG, ELANE, and MPO, which are mainly released by neutrophils, contribute to exacerbation and prolongation of inflammation. To verify the differential expression of CTSG, ELANE, and MPO proteins revealed by proteomic analyses (Figure 5D), we conducted immunohistochemistry staining for these three enzymes in sections of lung tissue from the five groups. Consistent with pathological results shown above, sections from healthy control lungs showed no neutrophil infiltration, and thus a low expression of all three enzymes. Five hours after LPS inhalation, CTSG, ELANE, and MPO protein levels were all significantly elevated in the lung, suggesting extensive neutrophil infiltration. Both low-dose and high-dose Andro-S treatment significantly decreased CTSG, ELANE, and MPO protein expression (Figures 6B,C), indicating alleviation of local inflammation and neutrophil infiltrations, results consistent with previous proteomic experiments.

## Discussion

In the present study, we induced pulmonary inflammation by exposing mice to aerosolized LPS for 30 min twice at a 2-h interval. This protocol resulted in severe lung injury characterized by histological changes and increased inflammatory cytokines in the BALF. Administration of a single dose of Andro-S after LPS inhalation alleviated pulmonary inflammation, resulting in a histological improvement in the lung and a reduction of TNFα protein levels in BALF. An immunohistochemical analysis of TNFα and IL6 expression in the lung also confirmed the anti-inflammatory effects of Andro-S. Besides, inhalation of LPS induced modest systemic inflammation, an effect that was strongly attenuated by injection of 10 mg/kg or 50 mg/kg Andro-S (Figure 1).

The murine model of LPS-induced lung injury is one of the most frequently used ALI models owing to its feasibility and high reproducibility (Matute-Bello et al., 2008). LPS can be administered through different routes, thereby indicating different responses in the lung. Systemic administration of LPS via intravenous injection initially injures the capillary endothelium, leading to apoptosis of endothelial cells, and ultimately causing systemic inflammation. Lung injury, characterized by changes in neutrophil deformability and entrapment of neutrophils in pulmonary capillaries and small numbers of neutrophils in the air space, become evident within 2–4 h after LPS injection. Another method for causing LPS-induced lung injury is local administration into the lung using an intratracheal catheter, which causes a robust increase in neutrophils in the air space. In the current study, we used a non-invasive and anesthesia-free approach for inducing ALI, by exposing animals to aerosolized LPS in an aerosol chamber. This method induced a more restricted and robust lung injury, characterized by a greater increase in inflammatory cytokines in BALF than in serum. These features may partly explain the limited effect of Andro-S on the cytokine levels in serum compared with that in BALF (Table 1).

Proteomics, as a tool for the global study of the whole cellular protein expression, is a powerful approach that has proven useful in drug discovery process because it has the potential to identify novel proteins involved in key biological processes in the cell that may serve as potential drug targets (Kopec et al., 2005). However, proteomic methods have rarely been used to unravel the mechanisms of action of andrographolide or its derivatives. In one of the few such studies, Hong et al. used a two-dimensional (2-D) electrophoresis proteomic approach to identify the key proteins involved in kidney dysfunction induced by administration of andrographolide sodium bisulfite (ASB) and to investigate the mechanism of adverse drug reactions to LianBiZhi (LBZ) (Lu et al., 2011). In the current study, we employed iTRAQ technology for the first time to identify potential drug targets of Andro-S. Five different treatment groups (*n* = 5 for each group) were used for iTRAQ-LC-MS/MS proteomic analysis: blank (saline + Andro-S 50 mg/kg), control, model (LPS + saline), low-dose treatment (LPS + Andro-S 10 mg/kg) and high-dose treatment (LPS + Andro-S 50 mg/kg). A total of 2234 proteins were quantified across all the 25 samples, 31 of which were identified as differentially expressed proteins based on strict filtration criteria. The expression profiles of some significantly changed proteins in our proteomic study align well with were consistent with the previously reported Andro-S-associated studies (Li et al., 2014; Wen et al., 2015). For example, increases in plasma MPO and S100A8/A9 levels have been reported in hand, foot, and mouth disease (HFMD) patients (Li et al., 2014; Wen et al., 2015), and Andro-S was demonstrated to be clinically effective against HFMD by reducing fever and inflammation (Li et al., 2013; Wen et al., 2015; Guoliang et al., 2017). Combination of subsequent GO annotation and PPI analysis, we selected ELANE, CTSG, and MPO, three enzymes mainly released by activated neutrophils, for validation by IHC in the lung tissues (Figure 6).

Upon inflammatory insult, neutrophils are the first immune cells to be recruited to the injury site providing a primary line of defense against bacterial infection. Although this process is essential for host defense and tissue repair, excessive activation of neutrophils can be deleterious (Lee and Downey, 2001; Jochen Grommes, 2011; Geering et al., 2013; Kruger et al., 2015), as highlighted by the fact that depletion of neutrophils in the mice reduces the severity of lung injury (Abraham et al., 2000; Looney et al., 2006).

Activated neutrophils release large amounts of cytokines and proteinases, which can exacerbate and prolong inflammation. ELANE and CTSG are two important serine proteinases released by neutrophils, and both can prolong and exacerbate inflammation by degrading extracellular matrix and anti-inflammatory proteins, promoting coagulation and thrombus formation, and activating pro-inflammatory signaling pathways. Administration of ELANE and CTSG induces lung inflammation (Lucey et al., 1985), whereas knockout of both ELANE and CTSG decreases mortality rate and lung injury in a murine sepsis model (Tkalcevic et al., 2000). Accordingly, many studies have sought to develop and evaluate specific inhibitors of ELANE or CTSG. Despite evidence for their protective effects in animal models, no inhibitors for ELANE have advanced to clinical trials except for sivelstat, which is approved for use in Japan and Korea (Perera and Jenne, 2012). Efforts to develop specific inhibitors for CTSG have trailed behind those for ELANE, and no effective CTSG inhibitors have entered clinical trials to date. However, preclinical studies have shown that a dual inhibitor of CTSG and chymase is effective in reducing inflammation in rat, sheep, and mice models (Maryanoff et al., 2012).

MPO, which also is mainly released by activated neutrophils, is a powerful pro-oxidative and pro-inflammatory enzyme. MPO protein levels and enzymatic activity in the BALF and tissue homogenates have been widely used as surrogate markers of neutrophil infiltration (Matute-Bello et al., 2011). Our study revealed that Andro-S protects the lung from LPS-induced ALI by reducing ELANE, CTSG, and MPO expression. But whether this effect is attributable to a reduction of neutrophils infiltration in the lung or direct inhibition of protein expression is not known and will require further investigation.

In conclusion, we demonstrated the anti-inflammatory effects of Andro-S against LPS-induced ALI and provided comprehensive profiles of proteins that are differentially expressed in murine lung tissues in response to LPS and Andro-S. Our results provide the evidence supporting the clinical potential of Andro-S for ALI treatment and shed light on its potential effector proteins/targets.

## Author contributions

FG, XL, ZS, HZ, and YW designed the experiments and wrote the manuscript. FG and ZS performed the animal experiments, pathological evaluations and data analyses. XJ calculated the acute lung injury score in a blinded manner. XL, HH, and JW performed the proteomic experiments. XL performed the data analyses. All authors contributed to manuscript revision, read and approved the submitted version.

### Conflict of interest statement

The authors declare that the research was conducted in the absence of any commercial or financial relationships that could be construed as a potential conflict of interest.
