# Supplementary figures and images for: Andrographolide Sulfonate Attenuates Acute Lung Injury by Reducing Expression of Myeloperoxidase and Neutrophil-Derived Proteases in Mice
Source: Front Physiol. 2018 Aug 17;9:939. doi: 10.3389/fphys.2018.00939 (PMC6107831; doi:10.3389/fphys.2018.00939)

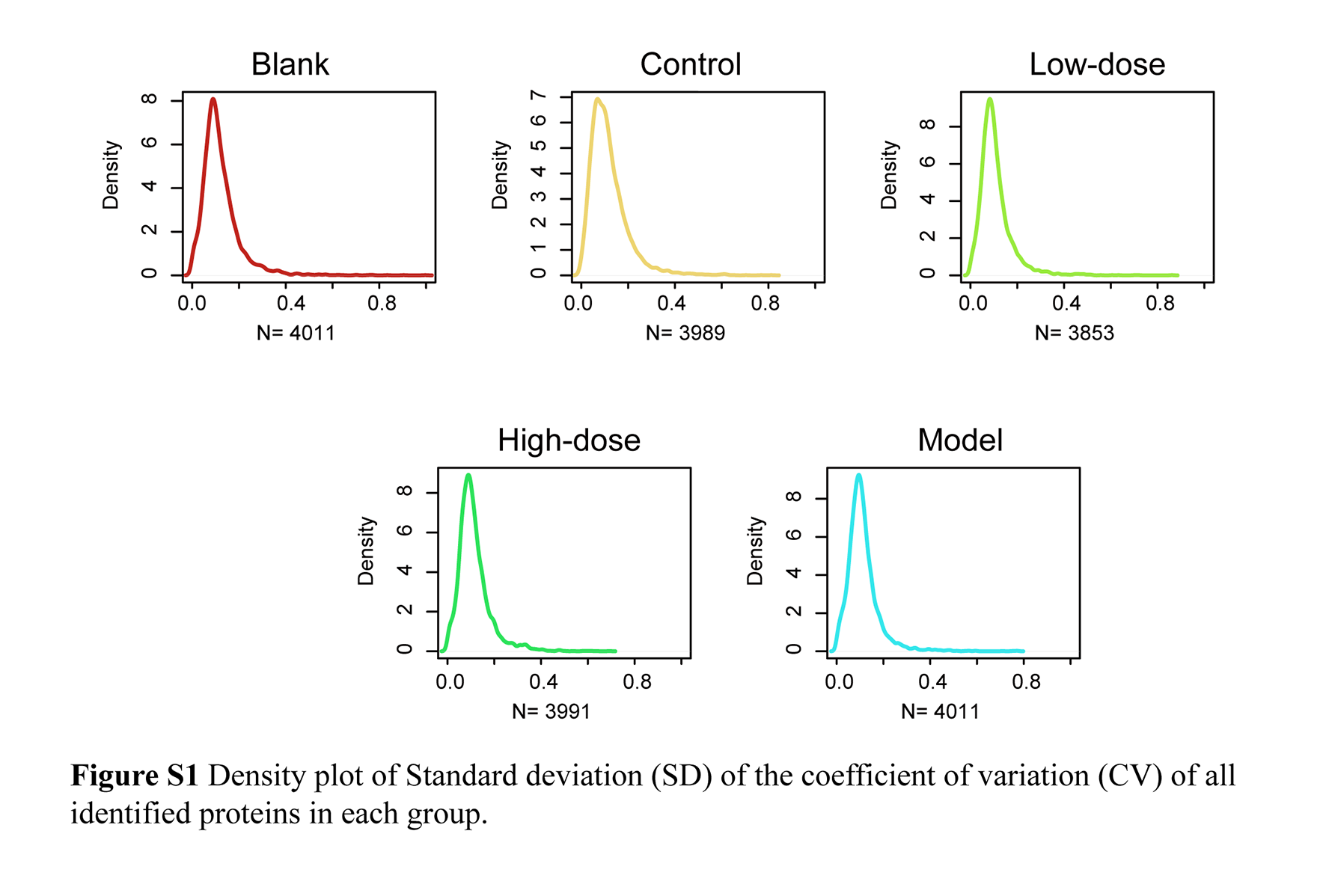

Supplement: Supplementary file 2 [file Image_1.TIF]

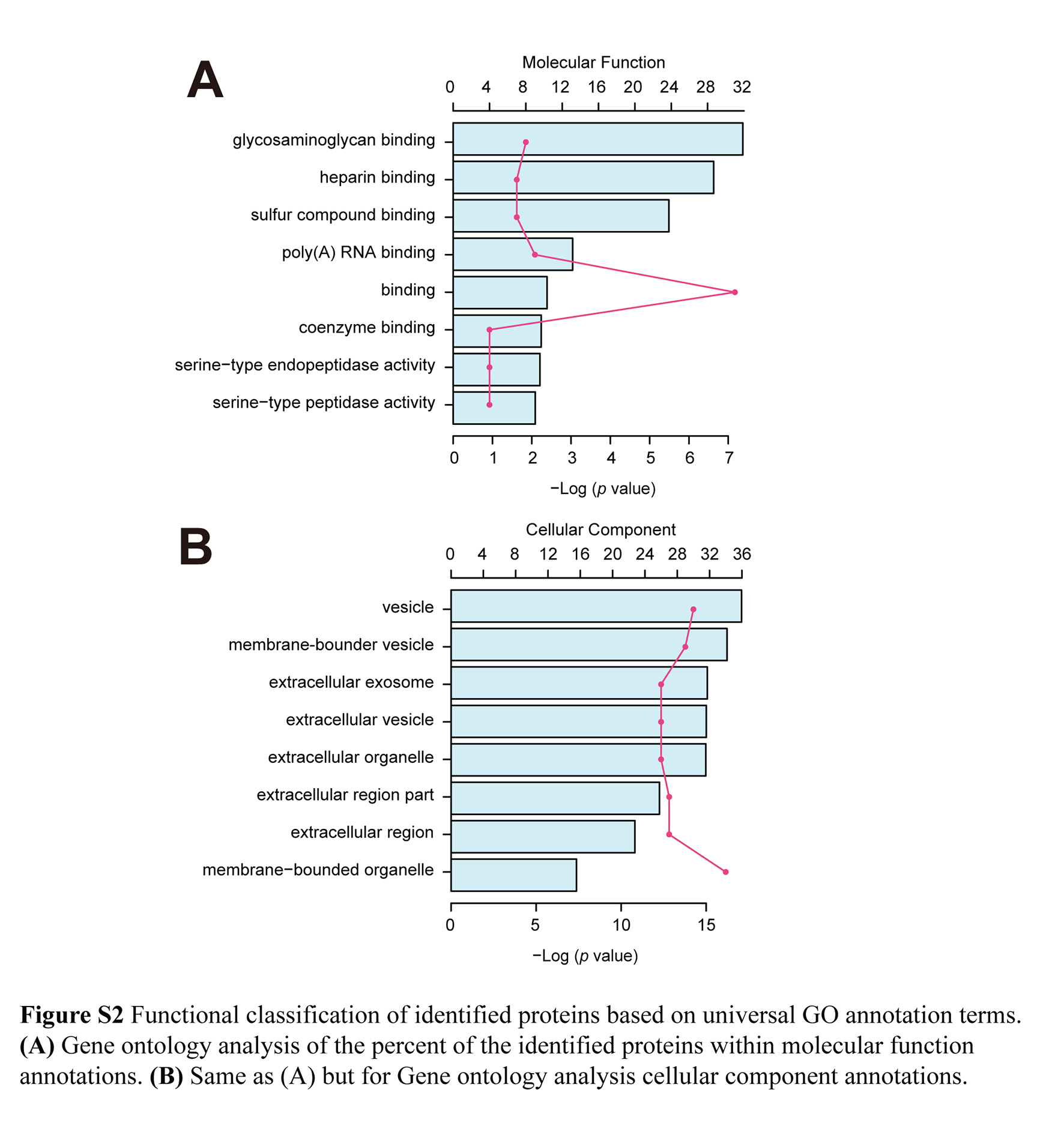

Supplement: Supplementary file 3 [file Image_2.TIF]
